# Pseudomonas aeruginosa infection correlates with high MFI donor-specific antibody development following lung transplantation with consequential graft loss and shortened CLAD-free survival

**DOI:** 10.1186/s12931-024-02868-1

**Published:** 2024-07-01

**Authors:** Levente Zoltán Bogyó, Klára Török, Zsuzsanna Illés, Anikó Szilvási, Bálint Székely, Anikó Bohács, Orsolya Pipek, Ildikó Madurka, Zsolt Megyesfalvi, Ferenc Rényi-Vámos, Balázs Döme, Krisztina Bogos, Balázs Gieszer, Eszter Bakos

**Affiliations:** 1 Department of Thoracic Surgery, Semmelweis University and National Institute of Oncology, Rath Gyorgy u. 7-9, Budapest, 1122 Hungary; 2 National Korányi Institute of Pulmonology, Koranyi Frigyes ut 1, Budapest, 1121 Hungary; 3 Hungarian National Blood Transfusion Service, Budapest, Hungary; 4 Department of Pulmonology, Semmelweis University, Budapest, Hungary; 5 Department of Physics of Complex Systems, Eotvos Loránd University, Budapest, Hungary; 6 Department of Thoracic Surgery, Comprehensive Cancer Center, Medical University of Vienna, Vienna, Austria; 7 National Institute of Oncology and National Tumor Biology Laboratory, Budapest, Hungary; 8 Department of Translational Medicine, Lund University, Lund, Sweden

**Keywords:** Lung transplantation, DSA, HLA, AMR, CLAD, Pseudomonas aeruginosa, BAL

## Abstract

**Background:**

Donor-specific antibodies (DSAs) are common following lung transplantation (LuTx), yet their role in graft damage is inconclusive. Mean fluorescent intensity (MFI) is the main read-out of DSA diagnostics; however its value is often disregarded when analyzing unwanted post-transplant outcomes such as graft loss or chronic lung allograft dysfunction (CLAD). Here we aim to evaluate an MFI stratification method in these outcomes.

**Methods:**

A cohort of 87 LuTx recipients has been analyzed, in which a cutoff of 8000 MFI has been determined for high MFI based on clinically relevant data. Accordingly, recipients were divided into DSA-negative, DSA-low and DSA-high subgroups. Both graft survival and CLAD-free survival were evaluated. Among factors that may contribute to DSA development we analyzed Pseudomonas aeruginosa (P. aeruginosa) infection in bronchoalveolar lavage (BAL) specimens.

**Results:**

High MFI DSAs contributed to clinical antibody-mediated rejection (AMR) and were associated with significantly worse graft (HR: 5.77, *p* < 0.0001) and CLAD-free survival (HR: 6.47, *p* = 0.019) compared to low or negative MFI DSA levels. Analysis of BAL specimens revealed a strong correlation between DSA status, P. aeruginosa infection and BAL neutrophilia. DSA-high status and clinical AMR were both independent prognosticators for decreased graft and CLAD-free survival in our multivariate Cox-regression models, whereas BAL neutrophilia was associated with worse graft survival.

**Conclusions:**

P. aeruginosa infection rates are elevated in recipients with a strong DSA response. Our results indicate that the simultaneous interpretation of MFI values and BAL neutrophilia is a feasible approach for risk evaluation and may help clinicians when to initiate DSA desensitization therapy, as early intervention could improve prognosis.

**Supplementary Information:**

The online version contains supplementary material available at 10.1186/s12931-024-02868-1.

## Background

Lung transplantation (LuTx) has a poor long-term outcome, with a current median post-transplant survival of 6.5 years [1], and 8.7 years for recipients surviving the first postoperative year [2]. Novel immunosuppressive regimens significantly eradicated acute graft-rejection events [3], yet chronic rejection manifesting as chronic lung allograft dysfunction (CLAD) represents the major complication in long term allograft survival, affecting ~ 50% of recipients at the first 5 years [1, 4]. No specific treatment is currently available to prevent or reverse CLAD, and the lack of appropriate biomarkers challenge the detection of the early and probably reversible phase of this condition [5].

The development of CLAD is multifactorial and several immune and non-immune related mechanisms have been suspected in its progression [6]. The generation of donor-specific antibodies (DSAs) against human leukocyte antigens (HLA) are common following LuTx [3, 7], with a wide range of reported incidence (12–47%)^8,9^. Previous studies analyzed the link between DSAs, graft loss and CLAD pathogenesis [6, 8, 9], however discrepancies often appear in clinical research [4, 10, 11, 9–13]. Not all DSAs are equally pathogenic, their level, HLA class or HLA-DQ specificity, complement-fixing traits, persistency or time of emergence all have been suspected to cause inconsistencies in clinical studies [14–16].

Respiratory tract infections often generate severe complications in immunosuppressed recipients that are now recognized risk factors of CLAD [17]. Continuous pathogenic provocation of the lungs, repetitive inflammatory episodes and impaired repair mechanisms lead to allograft deterioration over time. Pseudomonas aeruginosa (P. aeruginosa) is commonly found in LuTx recipients and aggravates tissue damage [18, 19]. Recently, P. aeruginosa colonization in respiratory specimens has been directly linked to the DSA response and shortened CLAD-free time [20].

In our present study we aimed to clarify the clinical impact of DSAs on graft survival and CLAD progression, and applied an MFI based risk stratification method to predict these outcomes. In search of factors contributing to DSA development we investigated the role of P. aeruginosa infection in BAL specimens. Additionally, we analyzed BAL immune cell ratios that correlated to DSA levels.

## Materials and methods

### Recipient cohort

All 116 recipients were transplanted by the Hungarian Lung Transplantation Program, between 12th December 2015– 7th August 2021, end of the follow-up time was 15th August 2022, median follow-up time was 735 days. 29 recipients who did not undergo DSA testing were excluded. Altogether 87 recipients have been analyzed. All patients were treated and managed similarly, according to standardized institutional protocol [21]. In brief, patients (*n* = 82 and *n* = 5, respectively) received induction therapy consisting of alemtuzumab (0.4–0.5 mg/kg) or polyclonal anti-thymocyte globulin (ATG) (2 mg/kg) as part of their immunosuppressive regimen. Following alemtuzumab induction, either a double combination therapy of tacrolimus and steroids was initiated, or a triple combination therapy of tacrolimus, mycophenolate mofetil, and steroids was applied [21–23]. Patients were closely monitored for CLAD regularly based on their DSA levels, and if indicated, predefined therapy was initialized before the appearance of clinical symptoms [24]. In cases of CLAD with BAL neutrophilia, we administered azithromycin at an immunomodulatory dose (250 mg three times a week) according to international recommendations [25–27]. Retransplantation was considered as a separate event in the outcome analysis. The outcomes were graft survival (death or retransplantation) and CLAD-free survival. When > 3000 MFI antibodies were detected pre-transplantation, the corresponding donor antigens were avoided. No standardized desensitization therapy was applied, *n* = 10 DSA positive recipients received plasmapheresis/intravenous immunoglobulin (IVIG) therapy.

### DSA detection

All diagnostic processes were conducted in accordance with the Hungarian National Blood Transfusion Service protocol. Anti-HLA-A, -B, -C, -DQ, or -DR antibodies were detected by LABScreen Single Antigen HLA Class I (LS1A04) and Class II (LS2A01) diagnostic tools (One Lambda, Thermo Fisher Scientific), following the manufacturer’s guidelines. In brief, 5 µl of LABScreen beads were incubated with 20 µl of test serum in a 1.5 ml micro-centrifuge tube for 30 min. Then, 1 ml of 1X wash buffer was added to each bead/serum solution tube and vortex, followed by centrifugation. Lastly, diluted PE-conjugated anti-human IgG was added to each tube, followed by the addition of PBS to the tubes. As for HLA genotyping in deceased donors, DNA was extracted from whole blood using the MagCore® Genomic DNA Whole Blood Kit and MagCore®Super instrument. Low-resolution HLA typing was obtained by performing DNA amplification and DNA-based, low-resolution typing for HLA-A, -B, - C, -DRB1, -DQB1 antigenic levels (Olerup SSP® HLA Typing Kits). Confirmatory typing was achieved by using LABType SSO A, -B, - C, -DRB1, -DQB1 Locus kits (One Lambda, Inc., Canoga Park, CA). The cutoff value for DSA positivity was > 1000 MFI. Immunodominant DSA defined as the highest MFI DSA for a given recipient.

### Defining CLAD and AMR

CLAD was defined according to the International Society for Heart and Lung Transplantation (ISHLT) guideline [6]: a persistent decline (> 20%) in measured forced expiratory volume (FEV1) value from the baseline value (mean of the best two postoperative FEV1 measurements taken > 3 weeks apart), and after exclusion of other causes of FEV1 decline. CLAD was definite if FEV1 decline lasted over 3 months. CLAD-free time was defined as the period between transplantation and the beginning of persistent FEV1 decline. AMR was classified based on ISHLT guidelines [28]. Recipients with or without DSA positivity, complement C4d staining and histology were classified as subclinical AMR. For clinical AMR additionally allograft dysfunction and clinical signs was measured by FEV1, radiology or by exclusion of confounding factors.

### BAL and microbiological analysis

For BAL ~ 120 ml 0.9% saline solution was applied in 40 ml fractions. Following suctioning, the fluid was analyzed for neutrophil percentages out of total inflammatory cells. BAL neutrophils below 25% were defined as “low” and above this threshold as “high”. Microbiological analysis for P. aeruginosa, Gram negative bacteria and fungi species have been performed. For active infection in BAL specimens a 10^3^ CFU/ml pathogen threshold has been determined.

### Statistical analysis

For data analysis Prism Graph Pad 9. and R version 4.2.1 was used. Ordinary one-way and two-way ANOVA were used to compare multiple groups. Contingency cohort analysis was used to calculate odds ratio, statistical significance and p-value were determined by Chi-square tests. Multiple MFI measurements from the same patient were treated as independent when investigating associations between AMR status, MFI value, HLA-DQ/class specificity and infections. Survival analysis was initially performed by fitting univariate Cox proportional hazard regression models for both graft and CLAD-free survival, while treating variables determined post-transplantation as time-dependent. For CLAD-free survival, events of death unrelated to CLAD were treated as censored observations. Multivariate Cox-regression models were fitted to the data for both outcomes with two sets of predetermined variables (AMR stages [time-dependent], presensitization, percentage of neutrophils in BAL specimens [time-dependent], infection [with P. aeruginosa, Gram negative bacteria or Candida species] [time-dependent] and DSA levels [time-dependent], presensitization, percentage of neutrophils in BAL specimens [time-dependent], infection). As AMR and DSAs are interconnected, we refrained from including both variables simultaneously in multivariate models of survivals. <0.05 p-values were considered statistically significant.

## Results

### Recipient cohort

A total of 283 sera from 87 recipients (Suppl. Table 1) have been analyzed. Most recipients were transplanted with chronic obstructive pulmonary disease (COPD) (47%) indication, followed by interstitial lung disease (ILD) (24%) and cystic fibrosis (CF) (20%). Of the cohort 36% tested DSA positive during the follow-up, and most recipients produced multiple antibodies. Of the recipients 19% developed class I, 32% class II specific DSAs and 49% developed DSAs against both classes (Suppl. Figure 1 A). The HLA-DQ specific DSAs were the most common and demonstrated significantly higher MFI values among all subtypes (MFI: 8527, median, *p* < 0.0001) (Suppl. Figure 1B-C). DSA production is an early event [29], the vast majority of DSAs were generated within the first 3 postoperative months (Suppl. Figure 1D).

### Antibody-mediated rejection (AMR) affects graft and CLAD-free survival

**Fig. 1:**
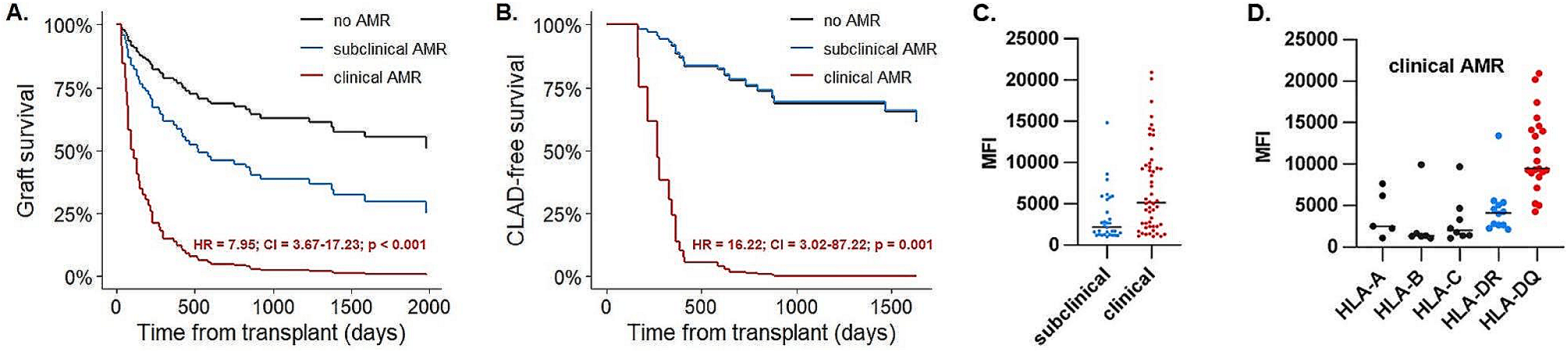
Analysis of AMR in LuTx recipients. (**A**) Expected adjusted graft survival curves for subpopulations of no AMR, subclinical AMR and clinical AMR calculated from the fitted univariate Cox-regression model with AMR as a time-dependent variable. The indicated hazard-ratio, confidence interval and p-value correspond to the clinical AMR vs. no AMR comparison. (**B**) Expected adjusted CLAD-free survival curves for subpopulations of no AMR, subclinical AMR and clinical AMR calculated from the fitted univariate Cox-regression model with AMR as a time-dependent variable. The indicated hazard-ratio, confidence interval and p-value correspond to the clinical AMR vs. no AMR comparison. (**C**) MFI values of DSAs associated with subclinical and clinical AMR. Each dot represents an individual DSA. MFI values in clinical AMR are significantly higher, (*n* = 85, median, one-way ANOVA *p* < 0.001). Black horizontal lines indicate mean MFI values within the groups. (**D**) The subtype specificity of DSAs causing clinical AMR, each dot represents an individual DSA. HLA-DQ was the most common type and had the highest MFI values, (*n* = 56, median, one-way ANOVA, *p* < 0.0001)

AMR is the central pathomechanism of DSA triggered graft damage, and it has been implicated as an independent risk factor for CLAD [14, 28, 30, 31]. Based on allograft dysfunction we divided our recipient cohort into no AMR, subclinical or clinical stage AMR groups and examined multiple outcomes. Recipients with clinical AMR demonstrated significantly worse graft survival when compared to the no AMR group (HR: 7.95, CI: 3.67–17.23, *p* < 0.001), while between subclinical and no AMR groups we could not detect a significant difference (HR: 2.04, CI: 0.92–4.53, *p* = 0.08) (Fig. 1A). Also, we examined the role of AMR in CLAD progression and observed significantly shorter CLAD-free survival in recipients with clinical AMR (HR: 16.22, CI: 3.02–87.22, *p* = 0.001) (Fig. 1B). Subclinical AMR did not differ from the no AMR group regarding CLAD-free time (HR: 0.98, CI: 0.22–4.25, *p* = 0.97). In contingency cohort analysis, clinical AMR significantly increased the probability of CLAD (OR: 7.8, CI: 1.67–39.92, *p* = 0.009), while subclinical AMR did not show a significant effect (OR: 1.12, CI: 0.27–4.48, *p* = 0.89). Examining the MFI values of DSAs in the subclinical and clinical AMR recipients we found significant differences (subclinical MFI: 3377 vs. clinical MFI: 6823, median, *p* < 0.001) (Fig. 1C). Within the clinical AMR cohort the frequency of the HLA-DQ subtype was increased and associated with a significantly higher MFI value when compared to other DSAs (MFI: 11,321, median, *p* < 0.0001) (Fig. 1D). Multivariate Cox regression models validated clinical AMR as an independent prognostic factor for both shorter graft survival (HR: 7.98, CI: 2.80-22.69, *p* < 0.001) and CLAD-free survival (HR: 34.79, CI: 4.14–292.30, *p* = 0.001) (Table 1).

**Table 1:** Multivariate Cox-regression models for graft and CLAD-free survival. Variables marked with an asterisk (*) were considered to be time-dependent. Statistically significant results are highlighted in bold. Results are represented as hazard ratio (HR), corresponding confidence interval (CI) and p-value

Variable	HR (CI)	*p*-value
**Effect of AMR on graft survival (concordance 74%)**		
AMR*		
No	Ref.	
Subclinical	1.89 (0.65–5.54)	0.244
** Clinical**	**7.98 (2.80-22.69)**	**< 0.001**
BAL neutrophil percentage*		
Low	Ref.	
** High**	**2.80 (1.18–6.67)**	**0.019**
Presensitization		
No	Ref.	
Yes	0.53 (0.19–1.50)	0.232
Infection (any kind)*		
Negative	Ref.	
Positive	1.13 (0.48–2.64)	0.781
**Effect of AMR on CLAD-free survival (concordance 73%)**		
AMR*		
No	Ref.	
Subclinical	1.84 (0.37–9.24)	0.458
** Clinical**	**34.79 (4.14–292.30)**	**0.001**
BAL neutrophil percentage*		
Low	Ref.	
High	3.65 (0.82–16.31)	0.090
Presensitization		
No	Ref.	
Yes	0.32 (0.04–2.61)	0.287
Infection (any kind)*		
Negative	Ref.	
Positive	0.84 (0.22–3.20)	0.799
**Effect of DSA MFI levels on graft survival (concordance 73%)**		
DSA*		
Negative	Ref.	
Low	0.62 (0.17–2.26)	0.470
** High**	**7.37 (2.61–20.82)**	**< 0.001**
BAL neutrophil percentage*		
Low	Ref.	
** High**	**2.85 (1.17–6.98)**	**0.022**
Presensitization		
No	Ref.	
Yes	0.99 (0.34–2.86)	0.984
Infection (any kind)*		
Negative	Ref.	
Positive	0.75 (0.31–1.80)	0.515
**Effect of DSA MFI levels on CLAD-free survival (concordance 61%)**		
DSA*		
Negative	Ref.	
Low	1.25 (0.24–6.37)	0.792
** High**	**22.04 (2.68-181.52)**	**0.004**
BAL neutrophil percentage*		
Low	Ref.	
High	2.41 (0.52–11.16)	0.259
Presensitization		
No	Ref.	
Yes	0.15 (0.02–1.42)	0.100
Infection (any kind)*		
Negative	Ref.	
Positive	0.98 (0.27–3.52)	0.976

### Graft- and CLAD-free survival in MFI stratified cohorts

**Fig. 2:**
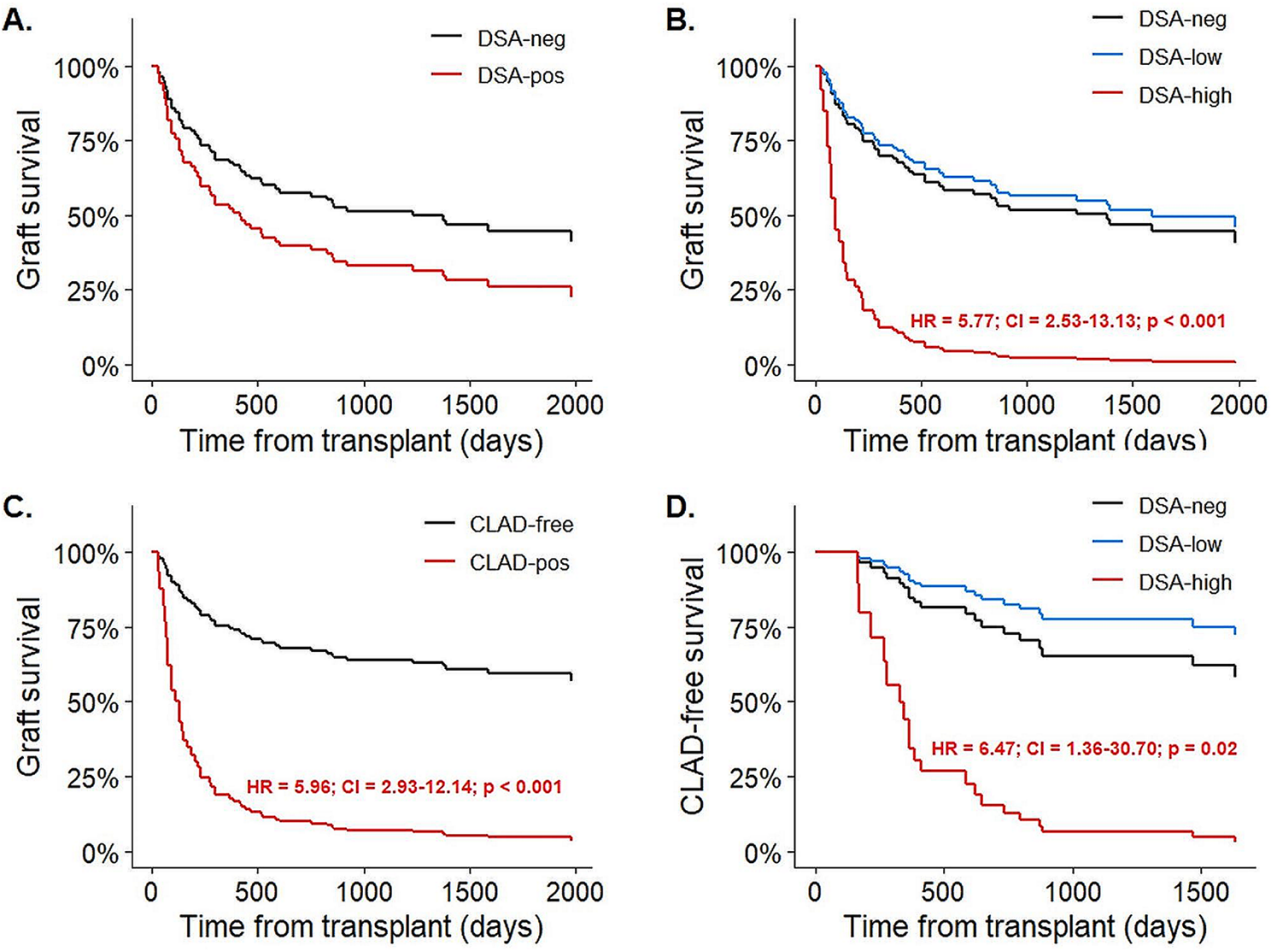
Univariate graft and CLAD-free survival analysis of LuTx recipients. (**A**) Expected adjusted graft survival curves for subpopulations with and without the presence of DSA calculated from the fitted univariate Cox-regression model with DSA as a time-dependent variable. (**B**) Expected adjusted graft survival curves for subpopulations DSA-high, DSA-low and DSA-neg calculated from the fitted univariate Cox-regression model with DSA as a time-dependent variable. The indicated hazard-ratio, confidence interval and p-value correspond to the DSA-high vs. DSA-neg comparison. (**C**) Expected adjusted graft survival curves for subpopulations that developed and did not develop CLAD during the follow-up period, calculated from the fitted univariate Cox-regression model with CLAD status as a time-dependent variable. The indicated hazard-ratio, confidence interval and p-value correspond to the CLAD-positive vs. CLAD-negative comparison. (**D**) Expected adjusted CLAD-free survival curves for subpopulations DSA-high, DSA-low and DSA-neg calculated from the fitted univariate Cox-regression model with DSA as a time-dependent variable. The indicated hazard-ratio, confidence interval and p-value correspond to the DSA-high vs. DSA-neg comparison

Investigating the effect of all DSAs on graft survival, we did not detect a significant difference between the sensitized versus non-sensitized groups (HR: 1.67, CI: 0.87–3.17, *p* = 0.12) (Fig. 2A). This finding prompted us to stratify our analysis based on MFI values. In search of the applicable MFI cutoff, we considered our data of DSAs triggering clinical AMR (MFI 6823) and reviewed a previous report of DSAs of clinical AMR recipients (MFI 7332) [32]. We added that HLA-DQ subtypes are overrepresented in clinical AMR that are accompanied with higher MFI values (11,321 MFI), which together pointed towards an average ~ 8000 MFI cutoff (Suppl. Figure 2A). Accordingly, we split the recipients into DSA-negative, DSA-low (1000–8000 MFI) and DSA-high (> 8000 MFI) groups. Using this stratification, we could demonstrate that high MFI DSAs were associated with significantly worse graft survival than recipients without or with low MFI DSAs (HR: 5.77, CI: 2.53–13.13, *p* < 0.0001 and HR: 6.64, CI: 2.24–19.67, *p* < 0.001) (Fig. 2B).

During the study period 28% of the recipients developed CLAD and showed a significant tendency for graft loss when compared to the CLAD-free group (HR: 5.96, CI: 2.93–12.14, *p* < 0.0001) (Fig. 2C). Among the MFI stratified groups, we detected a very strong association between high MFI DSAs and shorter CLAD-free survival when compared to DSA-negative and DSA-low cohorts (HR: 6.47, CI: 1.36–30.70, *p* = 0.02, HR: 10.82, CI: 1.45–80.67, *p* = 0.02). On the other hand, the DSA-low and DSA-negative groups did not differ significantly (HR: 0.60, CI: 0.14–2.62, *p* = 0.49) (Fig. 2D). Contingency cohort analysis revealed an 8.6 odds ratio (CI: 1.79–43.63, *p* = 0.006) for the DSA-high cohort to develop CLAD compared to the DSA-negative group, while the same calculation did not show a significant correlation for the DSA-low recipients (OR: 0.92, CI: 0.23–4.39, *p* = 0.9). Examining the grade of CLAD across the DSA stratified cohorts we did not observe higher grade in the DSA-high recipients, implying that DSAs impact onset time rather than the severity of CLAD (Suppl. Figure 2B). Additionally, the > 8000 MFI DSAs were predominantly class II (86%) and HLA-DQ (76%) specific, while in the DSA-low group 43% and 32% respectively (Suppl. Figure 2C-D). Analyzing the broad HLA mismatch scores of the DSA stratified groups we could not detect a difference that could explain the high HLA-DQ incidence in the DSA-high recipients (Suppl. Figure 2E). Multivariate Cox regression verified DSA-high status as independent prognostic factor for shortened graft- (HR: 7.37, CI: 2.61–20.82, *p* < 0.001) and CLAD-free (HR: 22.04, CI: 2.68-181.52, *p* = 0.001) survival (Table 1).

### Pseudomonas aeruginosa infection correlates with the DSA response

**Fig. 3:**
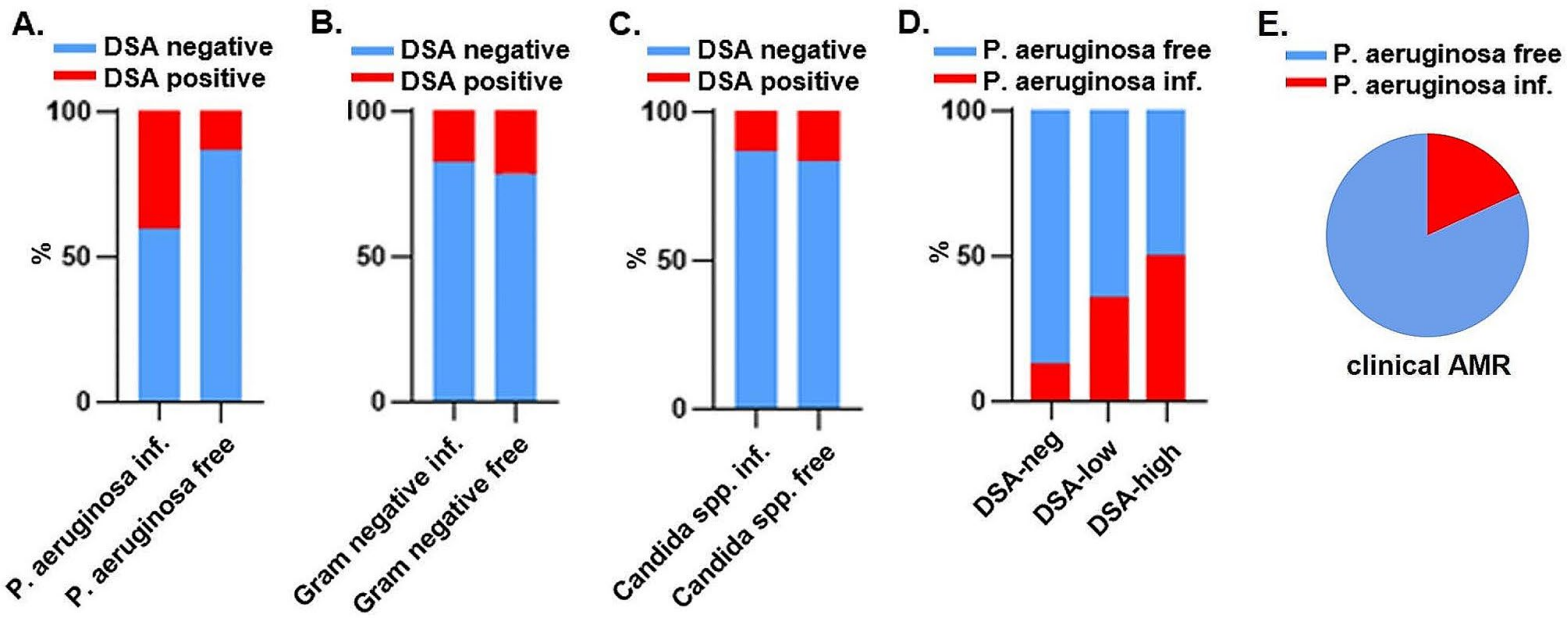
Pseudomonas aeruginosa infection correlates with DSA development. **A-C**. The percentages of P. aeruginosa (13% vs. 40.5%), Gram negative bacteria (17.6% vs. 21.4%) and Candida spp. (13.2% vs. 16.7%) infections in DSA negative and DSA positive cohorts. *n* = 83. **D**. P. aeruginosa infection percentages in DSA-neg, DSA-low and DSA-high recipient groups. DSA-high recipients show increased infection rate. *n* = 83. **E**. Pie chart represent the percents of P. aeruginosa infection (18%) or P. aeruginosa free samples (82%) in recipients where the symptoms of clinical AMR and Pseudomonas testing overlapped within 2 weeks, *n* = 11

P. aeruginosa colonization in respiratory specimens has been recently linked to DSA development [20]. Therefore we analyzed this phenomenon in our MFI stratified recipients, however we distinguished infection from colonization and used BAL specimens that were taken in close time proximity to DSA testing. To ensure that the effect is specific to P. aeruginosa, other Gram negative bacteria (Klebsiella pneumoniae, Klebsiella oxytoca, Escherichia coli, Acinetobacter baumannii, Achromobacter xylosoxidans, Citrobacter freundii, Stenotrophomonas maltophilia) and Candida species (C. albicans, C. crusei, C. glabrata) were analyzed simultaneously. In case of P. aeruginosa among the DSA-positive cohort 40.5% of BAL specimens tested positive for infection, while among the DSA-negative cohort only 13%, that is a ~ 3 fold increase (Fig. 3A). In case of Gram negative bacteria and Candida spp. similar percentages were found when comparing the DSA negative and DSA positive cohorts (21.4% vs. 17.6%, and 13.2% vs. 16.7%, respectively) (Fig. 3B-C). Contingency cohort analysis verified significant association between DSAs and P. aeruginosa infection (OR: 4.5, CI: 1.51–13.77, *p* = 0.0042), but not with other examined pathogens (Gram negative bacteria: OR: 0.79, CI: 0.23–2.58, *p* = 0.68: Candida spp.: OR: 0.76, CI: 0.27–2.36, *p* = 0.64) (Suppl. Table 2). In the DSA-negative cohort only 15.2% of the BAL samples were positive for P. aeruginosa infection, positivity increased to 30.8% and 53.3% in DSA-low and DSA-high recipients, respectively (Fig. 3D). The correlation was significant (DSA-low: OR: 3.75, CI: 1.07–12.36, *p* = 0.024; DSA-high: OR: 6.67, CI: 1.78–27.48, *p* = 0.0049), and did not manifest in case of other pathogens (DSA-low/Gram negative bacteria: OR: 0.58, CI: 0.16–2.22, *p* = 0.45; DSA-high/Gram negative bacteria; OR: 1.22, CI: 0.3–5.82, *p* = 0.79; DSA-low/Candida: OR: 0.63, CI: 0.17–2.25, *p* = 0.5; DSA-high/Candida: OR: 1.25, CI: 0.24–5.55, *p* = 0.79) (Suppl. Table 2). Previously we showed that clinical AMR is evident in DSA positive recipients. To ensure, that the clinical manifestation is related to DSAs and not P. aeruginosa infection we analyzed the overlap of clinical AMR and P. aeruginosa within a strict 2 weeks testing period. Indeed, when clinical AMR presented in recipients 82% of them was P. aeruginosa free, suggesting that clinical AMR is inherently DSA related, and Pseudomonas infection correlates to DSA emergence but not clinical AMR (Fig. 3E).

Of note, in univariate, time-dependent settings, none of the above investigated infections (or the aggregated presence of any of them) influenced either graft or CLAD-free survival in a significant manner.

### BAL neutrophilia correlates with DSA status

**Fig. 4:**
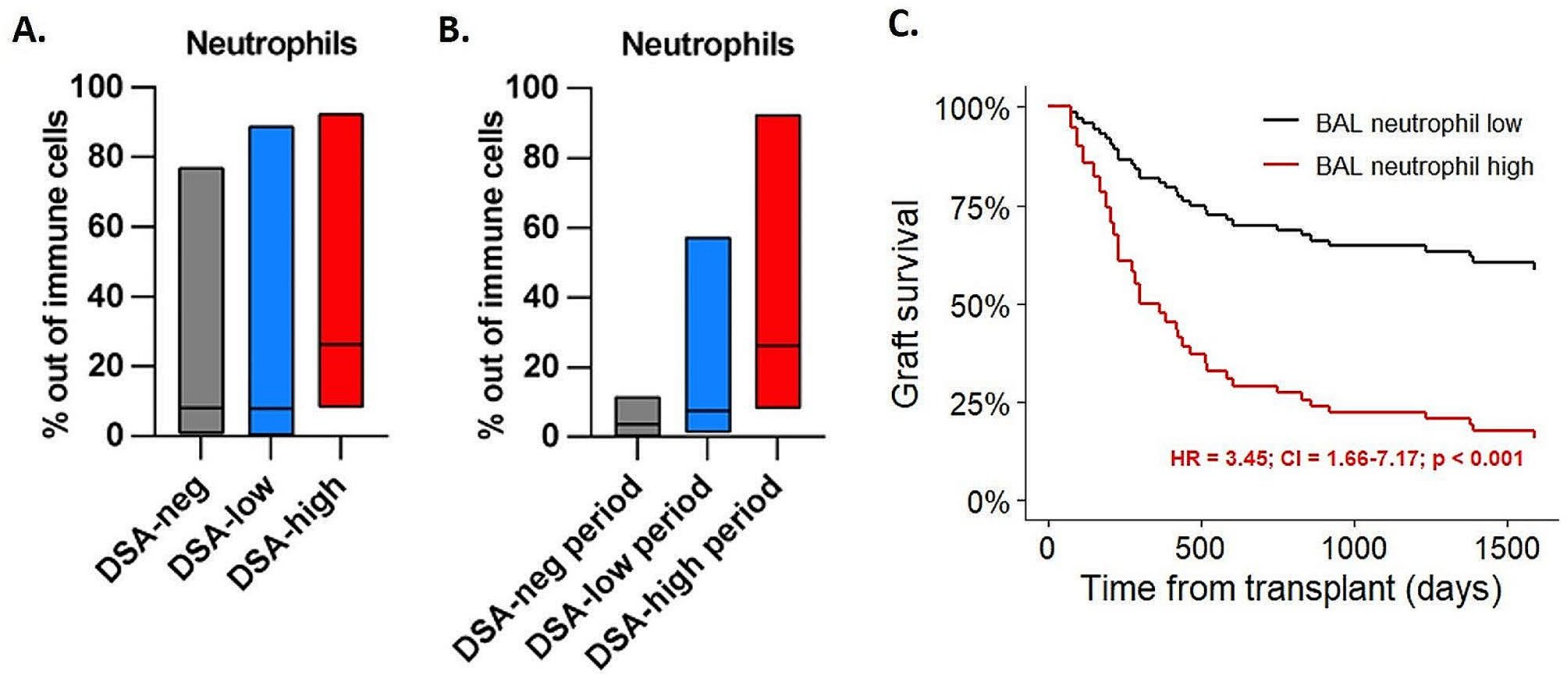
BAL immunophenotyping of LuTx recipients. (**A**) The % of neutrophils in BAL samples of DSA-neg, DSA-low and DSA-high recipients. Neutrophils in DSA-high recipients showed significant result, *p* < 0.001. (**B**) The % of neutrophils in BAL samples of DSA-high recipients taken at their DSA-neg, DSA-low and DSA-high clinical periods, *p* < 0.006. (**C**) Expected adjusted graft survival curves for subpopulations of high vs. low percentages of neutrophils in BAL specimens calculated from the fitted univariate Cox-regression model with neutrophil percentage as a time-dependent variable

In search of additional clinically relevant factors that correlate with DSAs we examined BAL immune cells in MFI stratified cohorts. When BAL immunophenotyping and DSA testing time overlapped, we found a significant increase in neutrophils in the DSA high group (DSA-negative: 8.04%, DSA-low: 7.9%, DSA-high: 26.3%, *p* < 0.001) (Fig. 4A). We further analyzed the BAL samples of only the > 8000 MFI DSA recipients, we split their data into DSA-negative, DSA-low and DSA-high clinical periods, and interestingly, we detected dynamic changes in their samples, showing that elevated MFI values correlated with increased BAL neutrophil ratios, most apparent in the DSA high clinical period (DSA-negative period: 3.7%, DSA-low period: 7.5%, DSA-high period: 26.3%, *p* = 0.006) (Fig. 4B). In a time-dependent model high BAL neutrophil ratios significantly decreased graft survival (HR: 3.45, CI: 1.66–7.17, *p* < 0.001) (Fig. 4C). In multivariate Cox regression models of AMR and MFI for graft survival BAL neutrophilia had an independently significant effect (HR: 2.80, CI: 1.18–6.67, *p* = 0.019 and HR: 2.85, CI: 1.17–6.98, *p* = 0.022, respectively) (Table 1). Of note, BAL neutrophilia did not influence significantly the occurrence of concurrent infections (*p* = 0.562; data not shown).

## Discussion

Allograft failure accounts for over 40% of deaths following LuTx [6]. DSAs are common during the postoperative period, nevertheless discrepancies are common when examining their roles in graft survival and CLAD progression [4, 10, 11, 9–13, 16]. In our current investigation we examined these outcomes in relation to the de novo DSA response when stratified by MFI levels and analyzed the role of P. aeruginosa infection in the humoral response. We identified high MFI DSAs and clinical stage AMR as independent prognostic factors for graft loss and poor CLAD-free survival. In addition P. aeruginosa infection correlates with DSA development, and BAL neutrophilia is a readily measurable sign of poor allograft prognosis.

The connection between AMR and DSAs are best characterized in kidney transplantation, with the least reports on lungs [3]. In our cohort recipients with clinical AMR had a strong correlation with graft loss and shortened CLAD-free time and using multivariate Cox-regression models we could identify clinical AMR as independent risk factor for both outcomes, while subclinical AMR could not be identified as such. DSAs eliciting clinical AMR had higher MFI values, and dominantly HLA-DQ specificity, that has been described as a relevant risk factors for AMR and graft damage [11, 33].

Analyzing graft survival based solely on DSA positivity we could not identify a difference compared to the DSA-negative cohort. While MFI is routinely used in the risk stratification pre-transplantation, the relevance of MFI by means of pathogenicity following LuTx has not been widely examined [14, 34]. Using a cutoff based on clinical data, we could clearly demonstrate that DSAs with high MFI have a profound effect on graft survival and MFI stratification is a relevant and widely available tool to evaluate future graft damage.

Applying DSA stratification, we could clearly demonstrate that high MFI DSAs shorten CLAD-free survival, while we could not detect the same effect in the DSA-low group. A previous report associated DSAs with a 2 fold CLAD risk [16], and a significantly shorter CLAD-free survival [9, 11]. We found a higher risk for CLAD in our cohort, and we hypothesize that the difference is enrooted in the MFI stratification method. Most > 8000 MFI DSAs were class II and HLA-DQ specific and showed similar traits as in previously reported studies, in which class II DSAs were shown to be risk factors for BOS [35], and 76% HLA-DQ specific DSAs induced CLAD [11]. HLA-DQ is the most immunogenic antigen, not only in the case of LuTx, but also in kidney and heart transplantation [11, 36, 37]. We suggest that the pathomechanism is enrooted in the inflammatory environment of the lungs, in which class II HLA expression may increase, as it was shown that inflammatory cytokines (INF-γ, TNF-α, IL-1b) elevate HLA class II expression on endothelial cells [33]. High cell surface HLA class II expression may trigger an elevated rate of different allorecognition pathways that ultimately lead to a strong DSA response and pulmonary damage [38, 39].

The factors triggering the DSA response are not completely clarified [22, 40]. Severe pulmonary infections frequently occur in immunosuppressed recipients. The tissue damage caused by pathogens and the impaired resolution are recognized risk factors for CLAD [17]. P. aeruginosa is commonly isolated from the airways of LuTx recipients, and its role in CLAD progression [19], and increased DSA risk have been reported [20]. Examining BAL specimens in our recipient cohort we found similar correlations. How P. aeruginosa provoke DSAs is unclear. Studies on CF patients showed that P. aeruginosa infected lungs have high B cell numbers [18]. The substantial tissue damage may act as potent proinflammatory signals for bystander B cell activation. Pathogenic and allo-antigen load may lead to the breakdown of tolerance in susceptible individuals. Furthermore, it has been shown that severe P. aeruginosa infection increased HLA-DR expression on airway epithelial cells that could enhance allorecognition mechanisms [17].

Several studies examined BAL immune cell compositions and its predictive value in rejection in LuTx recipients [41–43]. Elevated BAL neutrophil ratios of LuTx recipients correlated with acute rejection episodes [44–47] and subsequent CLAD progression [31, 42, 44]. Associating serum DSA levels to BAL cellularity we found profound BAL neutrophilia in recipients with high MFI DSAs. What we found particularly interesting is that BAL neutrophilia shifted dynamically in recipients when analyzed in different clinical periods based on DSA level changes. Additionally, BAL neutrophilia had a clear impact on graft loss. We hypothesize that using serum DSA and BAL data simultaneously may provide a unique tool to predict outcome, however a comprehensive and larger cohort analysis is needed to draw such conclusions.

Our study has limitations. This is a single center analysis and based on a limited number of recipients. The retrospective nature of the study may confound certain results and the clinical approach inherently left underlying mechanisms hypothetical. MFI is a semiquantitative measure of DSA levels and the lack of standardized diagnostic protocols may alter DSA cutoff results across different centers [14]. Moreover, serum DSA levels do not reflect the fraction of antibodies deposited in the lungs, which may falsely lead to reduced MFI values.

Nevertheless, few questions remain unclear and yet to be addressed in future studies. Unknown whether DSAs are important in the initiation or the progression phase of CLAD, which could have therapeutic consequences as when to start desensitization therapy. Our result on CLAD grade favors the former concept. Whether the risk of P. aeruginosa to enhance the humoral response is a causality or a bystander effect calls for further investigation.

## Conclusion

DSAs emerge shortly after LuTx, while consequential graft loss or CLAD follow in relative delay and the time between DSA detection and outcome may be sufficient to apply therapy. However, since all desensitization protocols bear side effects and significantly elevates the probability of infections, we suggest that considering MFI and BAL neutrophilia as prognostic factors may be beneficial to certain recipients and could guide clinicians to the right point when aggressive intervention is indicated.

### Electronic supplementary material

Below is the link to the electronic supplementary material.

Supplementary Material 1

Supplementary Material 2

Supplementary Material 3

Supplementary Material 4

## Data Availability

The datasets used and/or analyzed during the current study are available from the corresponding author upon reasonable request.

## Abbreviations

AMR: Antibody-mediated rejection
ATG: Anti-thymocyte globulin
BAL: Bronchoalveolar lavage
BOS: Bronchiolitis obliterans syndrome
CF: Cystic fibrosis
CI: Confidence interval
CLAD: Chronic lung allograft dysfunction
COPD: Chronic obstructive pulmonary disease
DSA: Donor-specific antibody
DSA-neg: DSA-negative
HLA: Human leukocyte antigen
HR: Hazard ratio
ILD: Interstitial lung disease
IVIG: Intravenous immunoglobulin
ISHLT: International Society for Heart and Lung Transplantation
LuTx: Lung transplantation
MFI: Mean fluorescent intensity
OR: Odds ratio
P. aeruginosa: Pseudomonas aeruginosa
RAS: Restrictive allograft syndrome
