# Supplementary figures and images for: Pseudomonas aeruginosa infection correlates with high MFI donor-specific antibody development following lung transplantation with consequential graft loss and shortened CLAD-free survival
Source: Respir Res. 2024 Jul 1;25:262. doi: 10.1186/s12931-024-02868-1 (PMC11218249; doi:10.1186/s12931-024-02868-1)

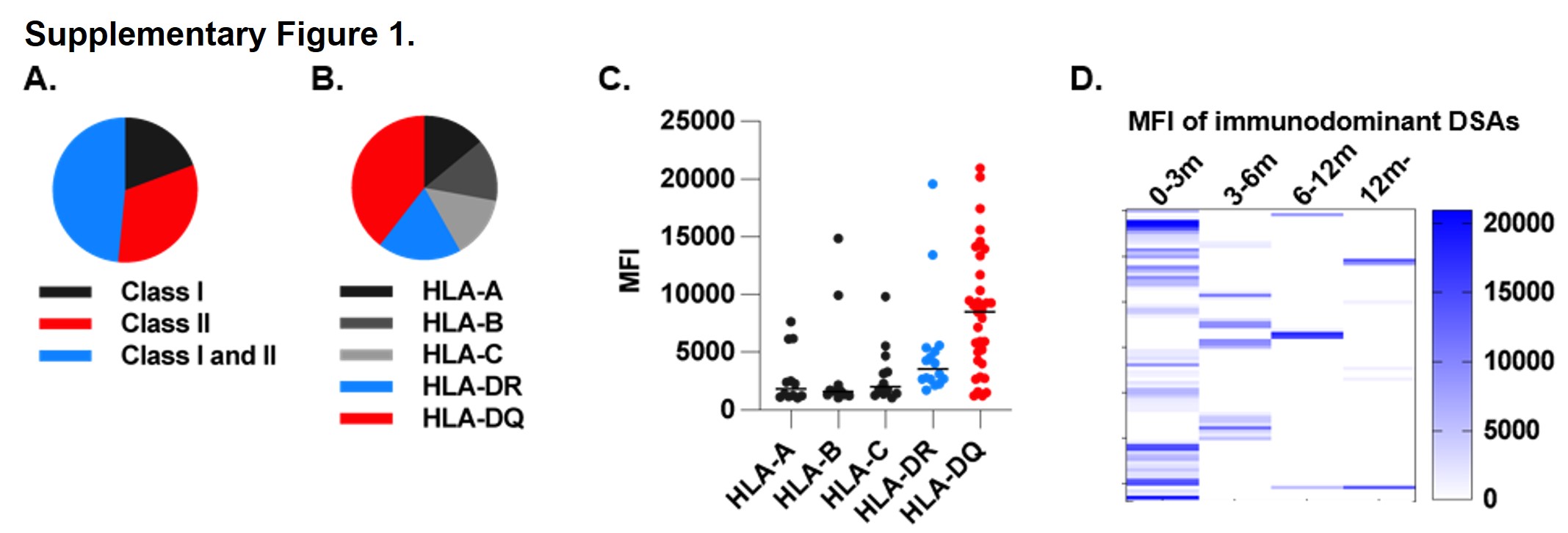

Supplement: Supplementary file 1 — Supplementary Material 1 [file 12931_2024_2868_MOESM1_ESM.jpg]

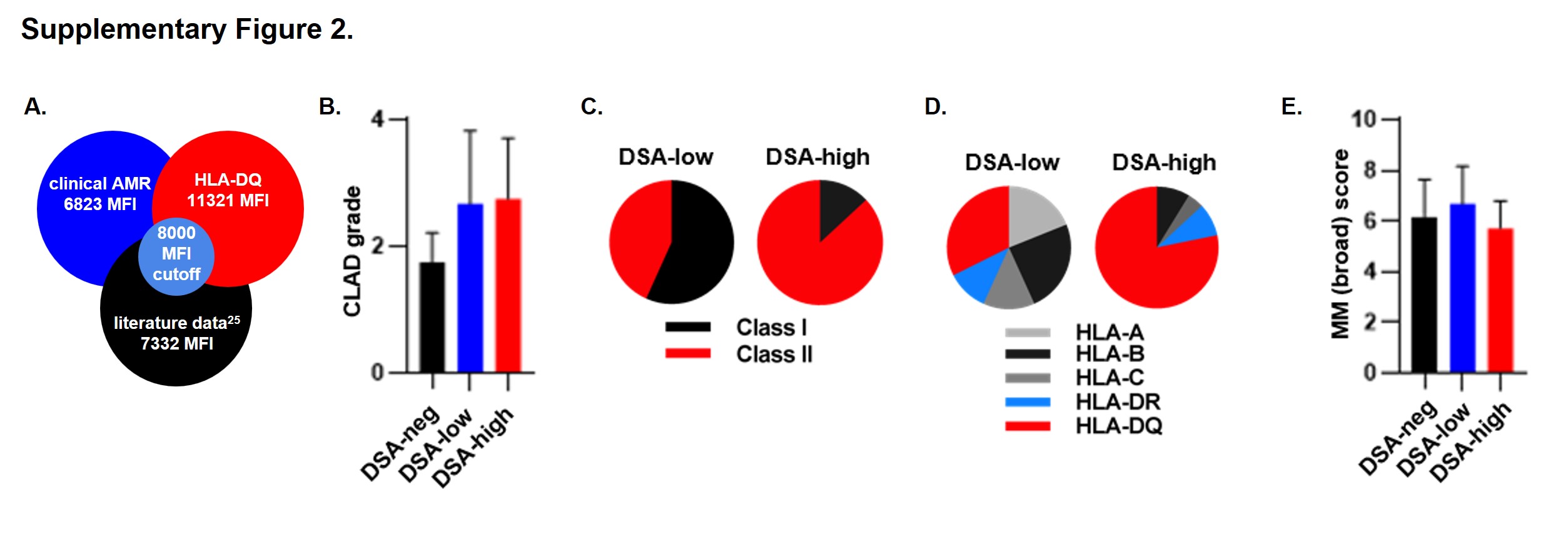

Supplement: Supplementary file 2 — Supplementary Material 2 [file 12931_2024_2868_MOESM2_ESM.jpg]
