# Supplementary material for: Pseudomonas aeruginosa infection correlates with high MFI donor-specific antibody development following lung transplantation with consequential graft loss and shortened CLAD-free survival
Source: Respir Res. 2024 Jul 1;25:262. doi: 10.1186/s12931-024-02868-1 (PMC11218249; doi:10.1186/s12931-024-02868-1)
Supplement: Supplementary file 3 — Supplementary Material 3 [file 12931_2024_2868_MOESM3_ESM.docx]

| **Supplementary Table 1.** |  |  |  |
| --- | --- | --- | --- |
|  | **All recipients** | **DSA negative** | **DSA positive** |
| **Recipients** | 87 | 56 (64%) | 31 (36%) |
| **Age at Tx** | 53 (22) | 55 (17) | 49 (34) |
| **Male/Female** | 43/44 | 31/25 | 12/19 |
| **Underlying disease** |  |  |  |
| **COPD** | 41 (47%) | 28 (67%) | 14 (33%) |
| **ILD** | 21 (24%) | 16 (76%) | 5 (24%) |
| **CF** | 17 (20%) | 9 (53%) | 8 (47%) |
| **PPH/IPAH** | 4 (5%) | 2 (50%) | 2 (50%) |
| **Bronchiectasia** | 2 (2%) | 1 (50%) | 1 (50%) |
| **Retransplantation** | 1 (1%) | 0 (0%) | 1 (100%) |
| **COVID-pneumonia** | 1 (1%) | 1 (100%) | 0 (0%) |
