# Supplementary material for: Pseudomonas aeruginosa infection correlates with high MFI donor-specific antibody development following lung transplantation with consequential graft loss and shortened CLAD-free survival
Source: Respir Res. 2024 Jul 1;25:262. doi: 10.1186/s12931-024-02868-1 (PMC11218249; doi:10.1186/s12931-024-02868-1)
Supplement: Supplementary file 4 — Supplementary Material 4 [file 12931_2024_2868_MOESM4_ESM.docx]

| **Supplementary Table 2.** |  |  |  |
| --- | --- | --- | --- |
|  |  |  |  |
|  | **OR** | **CI** | **p value** |
| **P. aeruginosa to DSA** | 4.54 | 1.52-13.77 | **0.0042** |
| **P. aeruginosa to DSA high** | 6.67 | 1.78-27.48 | **0.0049** |
| **P. aeruginosa to DSA low** | 3.75 | 1.07-12.36 | **0.024** |
| Gram negative bacteria to DSA | 0.79 | 0.23-2.58 | 0.68 |
| Gram negative bacteria to DSA low | 0.58 | 0.16-2.22 | 0.45 |
| Gram negative bacteria to DSA high | 1.22 | 0.3-5.82 | 0.79 |
| Candida spp. to DSA | 0.76 | 0.27-2.36 | 0.64 |
| Candida spp. to DSA low | 0.63 | 0.17-2.25 | 0.5 |
| Candida spp. to DSA high | 1.25 | 0.24-5.55 | 0.79 |
